# The Lymphatic System of the Human Body—Its Function—and the Identity of the Force Stored up in the Contents of the Thoracic Ducts, with the so Called Vital Force or Forces

**Published:** 1871-07

**Authors:** Z. C. McElroy

**Affiliations:** Zanesville, O.


ART. II.—*The Lymphatic System of the Human Body—its function—and the identity of the force stored up in the contents of the thoracic ducts, with the so called vital force or forces.*

BY Z. C. MCELROY, M. D., ZANESVILLE, O.

Recent efforts in influential medical circles to revive and re-instate in professional and public confidence, the now almost obsolete remedial measure of venesection, and the responses occasionally made eleswhere, induced me, some time since, to take up the subject for investigation. My purpose was to ascertain whether venesection was ever a proper remedial measure, and if so, whether there were any exact indications for its employment. In determining on a plan to be pursued in the investigation, from the many suggested to my mind, the following was adopted. To interrupt a practitioner, at the moment he was about opening a vein, and ask him the following questions:—"What is human blood? What is the relation the blood of this person bears to his, or her physical body? What end do you expect to attain by taking the blood from this person? Is anything known of how this end will be accomplished? Have you any exact indications to guide you in the use of the lancet?"

My design was to ascertain, if possible, whether these questions could be answered with the brevity and comprehensiveness required under such circumstances.

To find a reply to the first question, What is human blood? the food going into my own mouth at one meal, was fixed on as the starting point; and the endeavor to follow it through its various chemical mutations to blood; believing that in that way a more correct solution could be reached than any other. All the necessary data were at hand in works on chemical physiology for forming definite conceptions of what really took place among the more obvious changes in the mouth and stomach. But beyond that I soon got into difficulty. And at the system of chyliferous absorbents my progress was completely arrested. Before entering on the investigation, I thought I knew all about the absorbent system, not only in the abdomen but elsewhere. But I was not long in ascertaining that my knowledge of either was very imperfect.

All the books on anatomy, histology, physiology, chemical phisiology, and chemistry of man in my library were soon piled up before me, and a diligent study and review of the whole absorbent system entered upon. To my very great surpise the more I studied what the books taught in regard to the functions of the lymphatic system, the less I seemed to know, or rather feel certain about. If the pepsin of the stomach converted co-agulable albumens or proteins into peptones, or non-co-agulable albumens; and as peptones were so nearly identical with blood albumen, why this complicated apparatus for taking them up, and conveying them through the thoracic duct into the blood stream? Why was chyle—fasting—so nearly identical with lymph from other parts of the body in physical appearance, as well as chemical composition? The caliber of the thoracic duct, it seemed to me, was too insignificant to convey into the blood stream any considerable proportion of the products of the digestion of the food required by an ordinary sized man in health. Why are the contents of the thoracic ducts added to the blood stream so near the right heart? The only conclusion I found it possible to reach was that here was a mystery which physiology had not yet cleared up.

And so, not discouraged, I once more returned to the beginning; studied the anatomy of the whole absorbent system over again, the character of its histological elements of structure; its very great complexity; the large number of ganglia, plexuses and glands, again fixed my attention, but to deepen the mystery in regard to their function. And the only conclusion reached was that the lymphatic system had other functions than those now assigned to it by physiology. But what were they?

The next conclusion reached was that the lacteals did not take up any part of the products of intestinal digestion. The volume of chyle—its chemical structure, together with the fact—general law as immutable as gravity—that each histological structure in the human body performed its functions at the expense of its molecular forms of substance, forced that conclusion on my mind. What they did take up in the so-called lacteal system, was precisely what the absorbents took up in other parts of the body. The absorbent system was, therefore, a unit in the body in regard to function, connected with the abdominal viscera and elsewhere, be that what it may.

Then, again, that it was concerned in gathering up certain portions of the material resulting from tissue disintegration in the performance of function, seemed to be certain beyond any doubt whatever. But that what it did gather and pass through so complex a system of ganglia, plexuses and glands, was simply in the interest of economy of material, seemed to me altogether incredible, though that was the teachings of physiology. So that was rejected, and the search for a more satisfactory solution of their function renewed.

My attention was now turned to pathology, as I felt certain that here I would get some positive information. The lymphatic system in disorder, the result, Scrofula, the Kings Evil, a Cachexia; a state of the solid structures wholly different from those of an ideal physiological human body. Hereditary, a sort of leprosy, stamping the possessor of an imperfect lymphatic system, as a peculiar person; never in full health, and the remedial arts powerless to wholly change or eradicate the "diathesis." Closely allied was the condition known as Tuberculosis, another cachectic, and irremediable condition.

Pathology thus taught me that there was some very close relationship between a perfect physiological human body, and perfect lymphatic glands. And here I might have stuck forever, but for another incident in one of my studies of life, for another and different purpose, and in which I now saw that my conclusions fell something short of the reality—or rather fell short of reaching a symmetrical unity. In a previous study the present year on the correlation of the physical and vital forces,* I had reached the conclusion that pepsin, for instance, in converting coagulable albumens, into peptone, or now coagulable albumens, did more, and the more was in perpetuating and reproducing the molecular forms of the stomach itself—that is, stored up the force in so much material as was required for the accomplishment of this object. Not long after this conclusion had been reached, Prof. Flint's† paper was diligently studied, with the result that my conclusion that pepsin performed this function was somewhat strengthened, as well as that the peculiar organic principles elaborated by other viscuses and textures were credited with similar duties. And the general conclusion heretofore reached, that each histological structure, in the act of decay, and the performance of function, stored up the force for its own reproduction and perpetuation in one or more of the chemical compound there formed, considered settled. And I was all the more satisfied that this conclusion was correct, because it was in harmony with the mode of the reproduction and perpetuation of annuals in the vegetable world. For the vegetable seed represented so much material as was necessary to store up the force and maintain the young plant up to the point of independent existence, under favorable conditions—that is, with the capacity to appropriate other and new material, not furnished by the parent plant—to the evolution of its own special forms of structure in the vegetable world; and that like its parent, when the means for its own production and perpetuation were provided, the plant was dead, or died soon after.

And dead, because its mission in organic life was ended, in that it had perfected the means for its own perpetuation and reproduction.

With this simpler analagous process for guidance, I once more returned to the complexity of the absorbent system of the human body, to renew my study of it, determined, if possible, to find out its function—for it had a function, and an important one—and as

* Chicago Medical Journal. May, 1871.

† New York Medical Journal, March, 1871.

its gatherings were added to the blood stream at a particular point, I could not answer the question what was human blood until I cleared up this mystery of the function of the lymphatic system.

Returning to the stomah, I was not long in reaching the conclusion that but an unimportant part of the products, if any, of the food digested in it found their way into the blood through the lacteals and thoracic duct. And as they could not get there by accident, the further conclusion reached that they were passed, as other matter wholly soluble in water, directly into the venous blood vessels. This, however, modern physiology teaches as to a part of the products. These products underwent some not well undestood changes in the liver through which they were passed on their way to the lungs. But it seemed probable to me that the acids employed in the stomach were satisfied with alkalies in the liver—but whatever were the changes occuring there, the new material thus modified was forwarded on its way to the right heart, and with it, just before reaching the right heart, were mingled the contents of the thoracic ducts—the material collected by the general lymphatic system, and elaborated by its complex system of plexuses and glands, whatever its uses, were added to the stream containing these recent products of digestion.

It gradually became more and more evident to me, that here was the proper place where the material, in which was stored up the force for carrying this new material up into the dignity of tissue forms, ought to be added, and that that was the character and functions of the material supplied by the thoracic ducts.

From the right heart, the material in which was stored up the force for the reproduction of normal molecular forms of structure, momentarily disintegrating in the performance of fuuction—with the new material for that purpose supplied by the stomach—were forwarded to the lungs, the seat of, perhaps, the most active chemical changes in the whole body. The contents of the blood stream arriving there were, 1st—Old venous blood, certainly charged with some of the results of tissue disintegration, to find exit from the body throug the nostrils. 2d—The material supplied by the thoracic ducts, containing so much material as was necessary to store up the force for carrying it with new material up to the dignity of tissue forms of structure. 3d—The new material from the stomachic and intestinal digestion of food; in the lungs a fourth element was added, viz: the inhaled gaseous atmosphere. Among the chemical changes occurring in the lungs, carbonic acid was certainly disengaged, and found exit from the body through the nostrils; while oxygen, and very probably, nitrogen, were united with these complex materials. Other, not well understood chemical changes take place, with a result left in no doubt, viz: The chemical union of these four ingredients to form ARTERIAL BLOOD which was returned to the left heart, to be from thence distributed to the capillaries, the actual seats of molecular disintegration and repair.

I now felt that I had reached the necessary data to give a brief, comprehensive and accurate reply to the question whose solution had occasioned this particular investigation. In endeavoring to formulate the answer, none could be found more comprehensive or so brief as that given in Gen. ix. 4. "But the flesh, with the life thereof, *which is* the blood, &c." What is human blood? The life of human flesh. That is, the material and force for the construction of the various viscera and tissues composing a human body. Brief and pertinent, and compared with the accumulated data in regard to it, physiological, pathological, chemical and dynamic—accurate and scientific, and accounting for all its results.

But in obtaining the solution of this query, I had reached other unexpected, not sought after, and much more important results. In a word, I had brought to light the means by which the stream of new material, required, and actually going into the human body, during life, was carried up to the moleculer forms of structure, capable of performing its various functions.

The complexity of the means corresponded with the complexity of the ends accomplished, yet withal, so simple, so natural, so necessary, and it may be added, so obvious. My only surprise was that it had remained so long undiscovered. And it was in harmony with the means and ends used to perpetuate simple forms of organic life, which point unmistakeably to the existence of similar provisions in the more complex life of mobile animals.

And it unites in harmony man's spiritual nature and material body. Corresponds scientifically and accurately with the inspired account of the creation. "And the Lord God formed man of the dust of the ground, and breathed into his nostrils the breath of life, and man became a living soul." Gen. 11:7. Precisely what occurs in each individual case of reproduction and multiplication of the human body to day. The forms of the body are built up during intra-uterine life, of material common to earth--the food of the mother--the dust of the ground—and by the earths forces. For life, or motion, has still to be communicated to each individual human form through the nostrils, with the same result as in the beginning—a living soul! The act of creation, worthy of a Creator, the unmistakeable evidence of creative power, was the combining materials common to earth into molecular forms of organic structure, capable of evolving the complex phenomena of the human body, mechanical, chemical, thermal, emotional, sensational, and physiological, and providing means for their perpetuation, reproduction and multiplication. An ideal Creator, and ideal molecular forms of structure created; invisible spirit and physical science united.

My subsequent investigations have only resulted in accumulating additional evidence in support of the conclusions reached, and confirming their importance.

The foregoing facts and inductions, it seems to me, warrant the following general conclusions:

1st—That physiology has, up to this time, wholly misapprehended the functions of the lymphatic system of the human body.

2d—That the molecular forms of structure of the human body in the act of momentary disintegration and performance of allotted function, stores up in one or more of the chemical compounds then formed, the force necessary for their own reproduction, and perpetuation, as well as general and special physical contours.

3d—That the lymphatic system is the special means for the collection of the material in which the force for the preservation and perpetuation of the human body, as well in minutest detail, as in aggregate, is stored up; adding it to the blood stream to accompany new material from the stomach and intestinal track, to the lungs, to be subjected to the change brought about by the gases of the atmosphere; and to combine the material and force to form arterial blood, so that when returned to the left heart, and from thence distributed to the seats of molecular repair and disintegration,

These processes may proceed without interruption or delay, either of which occurring, life and function would soon be impaired or wholly arrested.

4th—That the force stored up in the contents of the thoracic ducts is identical with that which is now known as the vital force or forces of the human body.

